# Salicylic Acid-Responsive Factor *TcWRKY33* Positively Regulates Taxol Biosynthesis in *Taxus chinensis* in Direct and Indirect Ways

**DOI:** 10.3389/fpls.2021.697476

**Published:** 2021-08-09

**Authors:** Ying Chen, Hua Zhang, Meng Zhang, Wenli Zhang, Ziqi Ou, Zehang Peng, Chunhua Fu, Chunfang Zhao, Longjiang Yu

**Affiliations:** ^1^Department of Biotechnology, College of Life Science and Technology, Institute of Resource Biology and Biotechnology, Huazhong University of Science and Technology, Wuhan, China; ^2^Key Laboratory of Molecular Biophysics Ministry of Education, College of Life Science and Technology, Huazhong University of Science and Technology, Wuhan, China; ^3^Hubei Engineering Research Center for Edible and Medicinal Resources, Wuhan, China

**Keywords:** SA signal, WRKY transcription factor, taxol biosynthesis, *TcERF15*, *Taxus chinensis* 3

## Abstract

Taxol is a rare secondary metabolite that accumulates considerably in *Taxus* species under salicylic acid (SA) and methyl jasmonate treatment. However, the molecular mechanism of its accumulation remains unclear. We investigated *TcWRKY33*, a nuclear-localized group I WRKY transcription factor, as an SA-responsive regulator of taxol biosynthesis. Overexpression and RNA interference of *TcWRKY33* confirmed that *TcWRKY33* regulates the expression of most taxol biosynthesis genes, especially *10-deacetylbaccatin III-10-O-acetyltransferase* (*DBAT*) and *taxadiene synthase* (*TASY*), which were considered as key enzymes in taxol biosynthesis. Transient overexpression of *TcWRKY33* in *Taxus chinensis* leaves resulted in increased taxol and 10-deacetylbaccatin accumulation by 1.20 and 2.16 times compared with the control, respectively. Furthermore, *TcWRKY33, DBAT*, and *TASY* were confirmed to respond positively to SA signals. These results suggested that *TcWRKY33* was the missing component of taxol biosynthesis that responds to SA. The sequence analysis identified two W-box motifs in the promoter of *DBAT* but not in the *TASY*. Yeast one-hybrid and dual-luciferase activity assays confirmed that *TcWRKY33* can bind to the two W-boxes in the promoter of *DBAT*, upregulating its expression level. Hence, *DBAT* is a direct target of *TcWRKY33*. Furthermore, *TcERF15*, encoding a *TASY* activator, also contains two W-boxes in its promoter. Yeast one-hybrid and dual-luciferase activity assays further confirmed that *TcWRKY33* can upregulate *TASY* expression through the activation of *TcERF15*. In summary, *TcWRKY33* transmits SA signals and positively regulates taxol biosynthesis genes in two ways: directly and through the activation of other activators. Therefore, *TcWRKY33* is an excellent candidate for genetically engineering regulation of taxol biosynthesis in *Taxus* plants.

## Introduction

Taxol, also known as paclitaxel, is a specialized metabolite originally isolated from *Taxus* species that functions as an anticancer drug (Wani et al., 1971; Schiff et al., 1979). Similar to other secondary plant metabolites, such as artemisinin and vinblastine, taxol is very rare *in vivo* (Kwak et al., 1995; White, 2008; Abdul Rahim et al., 2018). More than 20 enzymes participate in the taxol biosynthesis pathway, making taxol biosynthesis more complex than the biosynthesis of artemisinin and camptothecin (Croteau et al., 2006). Thus, studies of the regulatory mechanism of taxol biosynthesis are beset with difficulties, resulting in inconsistency between commercial supply and the clinical requirements for taxol.

Salicylic acid (SA) is an important endogenous hormone in plants. It is involved in the regulation of the biosynthesis of secondary metabolites including terpenoids, alkaloids, and flavonoids (Kang et al., 2004; Tounekti et al., 2013). In suspension cultures of *Taxus chinensis* var. *mairei*, the addition of 20 mg/L of SA induces taxol biosynthesis (Wang et al., 2007). Miao et al. (2000) reported that the addition of 0.1 mg/L of SA can enhance the production of taxol by up to 3-fold. SA also increases the concentrations of 10-deacetylbaccatin (10-DAB) and baccatin III (Miao et al., 2000). Furthermore, many transcription factors function by responding to SA signals. PtrWRKY73, OsWRKY77, and BcWRKY46 respond to SA signals during defense responses (Wang et al., 2012; Lan et al., 2013; Duan et al., 2015). However, SA-induced transcription factors that regulate taxol biosynthesis remain to be discovered.

Only a few transcription factors are known to regulate the expression of taxol biosynthesis genes (Zhang et al., 2018b; Cui et al., 2019). Li et al. (2013) reported that the group I WRKY transcription factor TcWRKY1 can bind two W-boxes in the promotor of *10-deacetylbaccatin III-10-O-acetyltransferase* (*DBAT*) to upregulate its expression in *T. chinensis*. Lenka et al. (2015) identified three bHLH factors, TcJAMYC1/2/4, showing potential for downregulating several taxol biosynthesis genes, such as *phenylpropanoyltransferase* (*BAPT*), by binding to G- and E-boxes. In addition, TcJAMYC2 and TcJAMYC4 induce reporter gene expression under the control of *taxadiene 5-alpha hydroxylase* (*T5H*) and *taxadiene synthase* (*TASY*) promoters, respectively (Lenka et al., 2015). Moreover, two ERF factors (TcERF12 and TcERF15) and one MYC2 factor (TcMYC2a) strongly regulate the *TASY* gene by interacting with GCC- and G-like-boxes, respectively (Zhang et al., 2015, 2018b). All these factors are focused on the methyl jasmonate (MJ) signaling transduction pathway and may therefore effectively regulate taxol biosynthesis. Currently, no transcription factors responding to SA have been reported. Hence, this regulatory mechanism should be investigated.

WRKY transcription factors belong to one of the largest transcription factor families in plants (Eulgem et al., 2000; Robatzek and Somssich, 2001). Several WRKYs respond to SA treatment and play comprehensive and important roles in diverse physiological processes. For example, PtrWRKY73, a group I WRKY transcription factor, responds to SA treatment and plays a positive role in plant resistance to biotrophic pathogens (Duan et al., 2015). The SA-induced OsWRKY77 transcription factor functions as a positive regulator of defense/pathogenesis-related genes and makes transgenic plants highly resistant to infection by pathogens (Lan et al., 2013). In Chinese wild *Vitis quinquangularis*, VqWRKY52 plays essential roles in the SA-dependent signal transduction pathway and is capable of enhancing hypersensitive response cell death triggered by microbial pathogens (Wang et al., 2017).

In this study, we evaluated the potential functions of the group I WRKY transcription factor *TcWRKY33* in the regulation of taxol biosynthesis. We confirmed its induction by SA treatment and performed overexpression and RNA interference experiments to determine its effect on the content of taxol and related taxanes. Expression levels of taxol biosynthesis genes were determined by using quantitative real-time PCR (qRT-PCR). We subsequently investigated the molecular regulation mechanism of *TcWRKY33* through yeast one-hybrid assays, dual-luciferase reporter systems, and evaluation of expression patterns. Our results broaden our understanding of the positive roles of *TcWRKY33* transcription factors in regulating the biosynthesis of taxol in plants.

## Materials and Methods

### Plant Hormone Treatment

*Taxus chinensis* cell line #48 was maintained in modified Gamborg's B5 medium (62# medium) (Zhang et al., 2018a). Six-gram long-term sub-cultured cells of *T. chinensis* were collected into 62# liquid medium at 125 rpm for 48 h dark period. Then, 0.1 mmol/L of MeJA or 2.5 mmol/L of SA was added into the medium, respectively. The samples were taken after 0, 1, 3, and 6 h and ground in liquid nitrogen.

### Total RNA Isolation, cDNA Synthesis, and Cloning of *TcWRKY33* Gene

Fresh leaves of *T. chinensis* were collected from the Western Nursery Garden of Huazhong University of Science and Technology, Vilnius, Lithuania. Harvest samples were ground in liquid nitrogen and used for the isolation of total RNA. Total RNA isolation was conducted by using the RNAprep Pure Plant Kit (TIANGEN, Beijing, China, Cat: DP441), and the first chain of cDNA was transcribed by using the RevertAid First Strand cDNA Synthesis Kit (Thermo, USA, Cat: K1621).

The sequence of *TcWRKY33* gene was obtained from the multi-omics analysis data. It was found to harbor two WRKY domains in its deduced amino acid sequences, and its full-length was amplified by using reverse transcription-PCR. The primers named *TcWRKY33*-1F/R are shown in Supplementary Table 1. After amplifying with the template of *T. chinensis* cDNA, PCR products were T/A cloned into the pMD18-T vector (TaKaRa, Dalian, China) and sequenced.

### Sequence Alignment and Phylogenetic Analyzing of *TcWRKY33*

BLAST search (http://www.ncbi.nlm.nih.gov/BLAST/) was used for the homology search from the Swiss-Prot database. ClustalW online (https://npsa-prabi.ibcp.fr/cgi-bin/npsa_automat.pl?page=/NPSA/npsa_clustalw.html) at the default setting was used to align and evaluate the percentage identity of *TcWRKY33* with known WRKYs. The alignment results were sent to ESPript 3.0 (http://espript.ibcp.fr/ESPript/cgi-bin/ESPript.cgi) for coloring (Robert and Gouet, 2014). The phylogenetic analysis was performed with a neighbor-joining method by using 1,000 bootstrap resamplings in MEGA 7.0.

### Subcellular Localization

The full-length *TcWRKY33* Open Reading Frame (ORF) without termination codon was amplified using the primers *TcWRKY33*-2F/R in Supplementary Table 1 and then inserted into pCAMBIA1300-35S-sGFP with *Sac*I and *BamH*I restriction sites, and then recombinant vector was transformed into GV3101. The onion epidermal cells with a dimension of 1 × 1 cm were infected by positive clones and controls (pCAMBIA1300-35S-sGFP) in 1/2 MS liquid medium at 25°C in dark for 2 days. The infected onion epidermal cells were then plated on the MS medium for 2 days at 25°C in darkness. The GFP fluorescence was observed by using the laser scanning confocal microscope FV1000 (OLYMPUS, Japan).

### Cloning, Activity Validation, and Promoter Analysis of the 5′-Flanking Sequence of *TcERF15* Gene

First, the Coding DNA Sequence (CDS) of *TcERF15* of *T. chinensis* was aligned with *Taxus baccata* genome sequences, suggesting that it was highly conserved in both *Taxus* spp. In addition, the 5′-flanking sequence of this gene of *T. baccata* was obtained. To obtain and validate the similarity of flanking sequences of this gene in *T. chinensis*, the forward primer was designed according to the homolog sequence of *T. baccata*, while the reverse primer was designed according to the ORF sequence in *T. chinensis* (Supplementary Table 1). After amplifying with the template of *T. chinensis* genomic DNA, PCR products were T/A cloned and sequenced. Only those that contain 5′-UTR Untranslated Regions and partial ORF sequence of *TcERF15* were considered as the 5′-flanking sequence in *T. chinensis*.

Then, the fragment of *TcERF15* was inserted into pBI121 with *Hind*III and *BamH*I restriction sites, so that the β-glucuronidase (GUS) reporter was under the control of the promoter of *TcERF15*. The construct was transformed into onion epidermis cells mediated by GV3101 affection (Zhang et al., 2018b). To test its promoter activity, GUS staining was conducted according to our previous report (Li et al., 2013).

Then, the 5′-flanking sequence was analyzed to locate basic *cis*-elements and W-boxes by using PlantCare (http://bioinformatics.psb.ugent.be/webtools/plantcare/html/), PLACE (http://www.dna.affrc.go.jp/PLACE/signalscan.html) and Nsite (http://www.softberry.com/berry.phtml?topic=nsite&group=programs&subgroup=promoter).

### Yeast One-Hybrid

For prey vectors, *TcWRKY33* was ligated into pGADT7-Rec2 with *EcoR*I and *BamH*I restriction sites, resulting in a *TcWRKY33*::pGADT7-Rec2 recombinant vector (Supplementary Table 1). For bait vectors, sense, and antisense of 6-bp fragments containing W-boxes from promoters that were quadrupled with cohesive ends of *EcoR*I and *Sac*I were artificially synthesized, then the double strand generated by annealing was linked with digested pHIS2.1 vector, resulting in a series of bait vectors (Supplementary Table 1). Finally, each bait vector was co-transformed with the prey vector into the yeast strain Y187, and then they were plated onto the SD/Leu-/Trp-/His-deficient medium with 20/40 mM 3-AT for 3–5 days. Each bait vector co-transformed with empty pGADT7-Rec2 was used as the negative control.

### Construction of Overexpression and RNAi Vectors

The full-length of *TcWRKY33* was amplified from *TcWRKY33*::pMD18-T vector using the specific primers *TcWRKY33*-3F/R in Supplementary Table 1 and inserted into the vector with *Nco*I restriction site to generate the overexpression vector *TcWRKY33*::pCAMBIA3302. For the RNAi vector, a 376 bp 3′-CDS fragment of *TcWRKY33* was forward and reversely inserted into the LH-FAD2-1390RNAi vector so that the two fragments and the FAD-RNAi fragment would generate a stem-loop in cells (Supplementary Table 1). Then, the recombinant vectors were transformed into LBA4404, and LBA4404 containing empty pCAMBIA3302 or LH-FAD2-1390RNAi vector was used as the control, respectively.

### Transient Transformation of *TcWRKY33* in *T. chinensis* Leaves

For transient transformations of *Taxus* leaves, the single colonies of LBA4404 containing empty vectors, *TcWRKY33*::pCAMBIA3302 and *TcWRKY33*::LH-FAD2-1390RNAi, were cultivated in 20 ml of LB medium (LB) until OD600 = 0.6–0.8, and then they were collected and resuspended with 20 ml of half-strength MS liquid medium containing 200 μM of acetosyringone. The fresh leaves were gathered from blade tips on the 10-year-old *T. chinensis* potted plants. After surface disinfection, small incisions were made with a sterile scalpel in the inner surface of the leaves to facilitate *Agrobacterium* infection. Then, the injured leaves were cultured in a shaker at 125 rpm for 12 h in the dark at 28°C. After that, the infected *T. chinensis* leaves were plated on the 1/2 MS solid medium for 24 h at 28°C in darkness. Finally, the part of leaves was placed into the liquid nitrogen and then stored in the fridge at −80°C for the following experiments, and the others were dipped into sterile water shaking at 120 rpm for 6 days to test the contents of the taxol and 10-DAB.

### Quantitative Real-Time PCR and High-Performance Liquid Chromatography

Quantitative real-time PCR was conducted as previously described (Zhang et al., 2015), and three fully independent biological replicates were obtained for the statistical analysis (Supplementary Table 1). Each experiment was conducted three times, and we used Student's *t*-test. The treated leaves were dried at 60°C for 24 h and crushed to 40 meshes, 0.1 g of powder was first weighed accurately and put into a 10-ml centrifuge tube, and then 3 ml of methanol were added. The extraction procedure was conducted by using an ultrasonic machine (Kunshan KQ-250E, China) for 30 min, centrifuged for 10 min at 8,000 r/min, and repeated three times, and the upper phase was collected and dried using N-Evap (Autoscience MTN-2800D, China). The taxane samples were diluted by high-performance liquid chromatography (HPLC)-grade methanol and then passed through membrane filters (0.22 μm) before HPLC detection. Each experiment was conducted in triplicate. The extraction compounds were analyzed using the Agilent 1290 HPLC system combined with a phenyl-C18 column (250 × 4.6 mm, 4.6 μm). Chromatographic separation was performed by the gradient elution process of mobile phase water (A) and acetonitrile (B) as follows: 0–10 min, 5–40% B; 10–15 min, 40–50% B; 15–30 min, 50–80% B; 30–35 min, 80–95% B. The flow rate was 1.0 ml/min with 20 μl of injection volume. In addition, the column temperature and the detection wavelength were kept at 30°C and 227 nm, respectively.

### Effector–Reporter Assays

For dual-luciferase activity assay, the original 383 bp *DBAT* promoter/715 bp *TcERF15* promoter and two progressive deletions removing the W_a_-box/W_1_-box and W_b_-box/W_2_-box were cloned with restriction sites of *Hind*III and *BamH*I and inserted into the pGreenII-0800-Luc vector to generate the reporter vector (Supplementary Table 1). Then, *TcWRKY33*::pCAMBIA3302 acted as the effector vector. Each reporter and effector were transiently expressed in *Nicotiana benthamiana* leaves *via Agrobacterium* tumefaciens strain GV3101. The infiltrated plants were grown in a greenhouse for 24 h in darkness and then grown under natural conditions for 2 days, and the luciferase activities were measured with a dual-luciferase reporter assay kit (Vazyme).

## Results

### *TcWRKY33* Is an SA-Responsive Factor

Salicylic acid and MJ enhance the production of taxol (Yukimune et al., 1996; Miao et al., 2000). WRKY transcription factors are important SA and MJ signaling regulators, and *DBAT* and *TASY* respond to SA and MJ signals (Zhang et al., 2017). Analyzing the hormonal response patterns of *TcWRKY33* will therefore enrich our understanding of the regulatory network of taxol biosynthesis.

**Figure 1:**
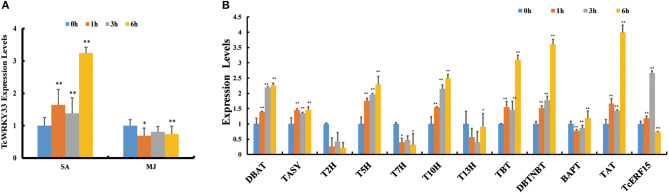
Induction of *TcWRKY33* by salicylic acid (SA) and methyl jasmonate (MJ) and the expression of taxol biosynthesis genes and *TcERF15* in SA treatment. **(A)** Relative expression of *TcWRKY33* in response to SA and MJ. **(B)** Relative expression of *TcERF15* and taxol biosynthesis genes in response to SA. Cells were harvested at different times (0, 1, 3, and 6 h) after treatment. Actin was used as an endogenous reference. The statistics method of *p*-value was Student's *t*-test. Asterisks indicate the significance, ^*^0.01 < *p* < 0.05, and ^**^p < 0.01.

Phytohormone treatment was conducted in *T. chinensis* cells and we measured the expression of *TcWRKY33* by using qRT-PCR. Expression of *TcWRKY33* was slightly higher at 1 and 3 h after SA treatment compared with that before treatment but increased by 3.24 times at 6 h after the induction of SA. However, *TcWRKY33* expression showed no obvious difference after MJ treatment. Hence, *TcWRKY33* responded significantly to the SA signal but not to MJ (Figure 1A). The response of *TcWRKY33* to SA was not immediate; accumulation of *TcWRKY33* transcripts after the induction of SA took time.

In addition to *TcWRKY33*, many taxol biosynthesis genes also responded to SA signals. Expression levels of *DBAT, TASY, T5H, 5-alpha-taxadienol-10-beta-hydroxylase* (*T10H*), *taxane 2-alpha-O-benzoyltransferase, 3*′*-N-debenzoyltaxol N-benzoyltransferase* (*DBTNBT*), and *taxadienol acetyltransferase* (*TAT*) were increased by 2.26-, 1.47-, 2.29-, 2.49-, 3.10-, 3.60-, and 3.99-folds, respectively, at 6 h after SA treatment compared with control (Figure 1B), indicating that these taxol biosynthesis genes respond positively to SA signals.

### *TcWRKY33* Is a Nuclear-Localized Group I WRKY Transcription Factor

**Figure 2:**
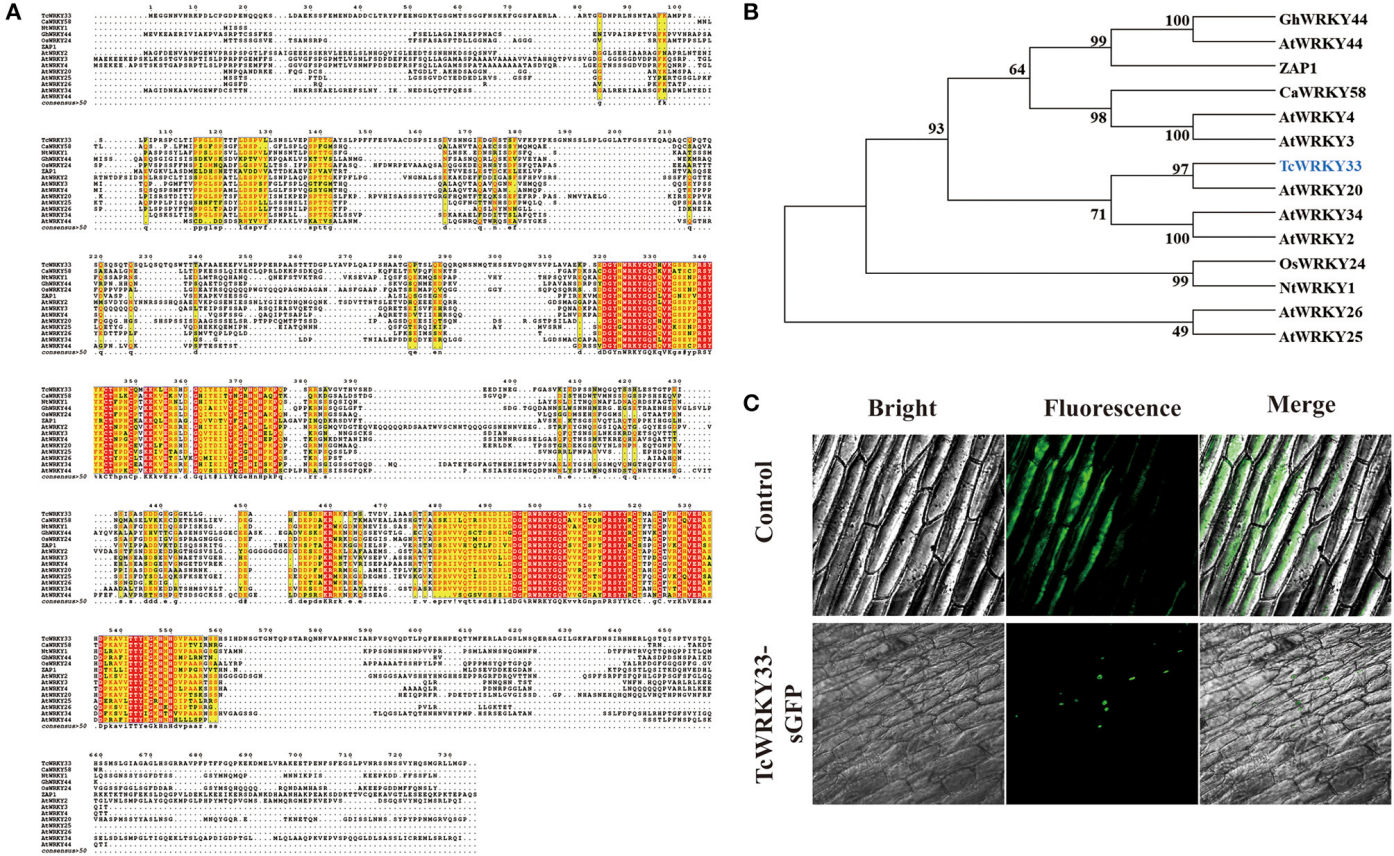
Sequence alignment, phylogenetic analysis, and subcellular localization of *TcWRKY33*. **(A)** Amino acid sequence alignment of *TcWRKY33* and other WRKYs in *Arabidopsis thaliana, Capsicum annuum, Nicotiana tabacum*, and *Oryza Sativa*. **(B)** Phylogenetic analysis of *TcWRKY33* and other WRKYs. *TcWRKY33* was signed in blue. **(C)** The fused protein of *TcWRKY33* and GFP was transformed into onion epidermal cells. GFP fluorescence was observed 2 days after infecting by using a laser scanning confocal microscope FV1000 (OLYMPUS, Japan) with 10 × 20 magnification.

An online Blast search of the SWISS-Prot database showed that *TcWRKY33* has the most similarity to the WRKY transcription factors, SUSIBA2 and AtWRKY20, and might thus function as an activator (Sun et al., 2003; Nagata et al., 2012). Sequence alignment revealed that the putative *TcWRKY33* contained two conserved WRKY domains in the N-terminus and C-terminus, followed by a Cys2/His2-type zinc-finger motif; it, therefore, represents a member of the group I WRKY transcription factors (Figure 2A). The *TcWRKY33* protein also contained an LSPLL motif, which is present in many transcription factors and cofactors and mediates interactions that activate or repress transcription (Plevin et al., 2005). The phylogenic analysis indicated that the *TcWRKY33* protein was closely related to the group I WRKY family members, sharing the highest similarity with AtWRKY20 (Figure 2B).

Subcellular localization in onion epidermal cells to verify the site of *TcWRKY33* activity revealed that *TcWRKY33* is a nuclear-localized protein (Figure 2C). Most transcription factors function in the nucleus; in particular, WRKY factors are DNA-binding proteins, suggesting their localization in the nucleus (Ciolkowski et al., 2008).

### *TcWRKY33* Upregulates Seven Genes Involved in Taxol Biosynthesis

**Figure 3:**
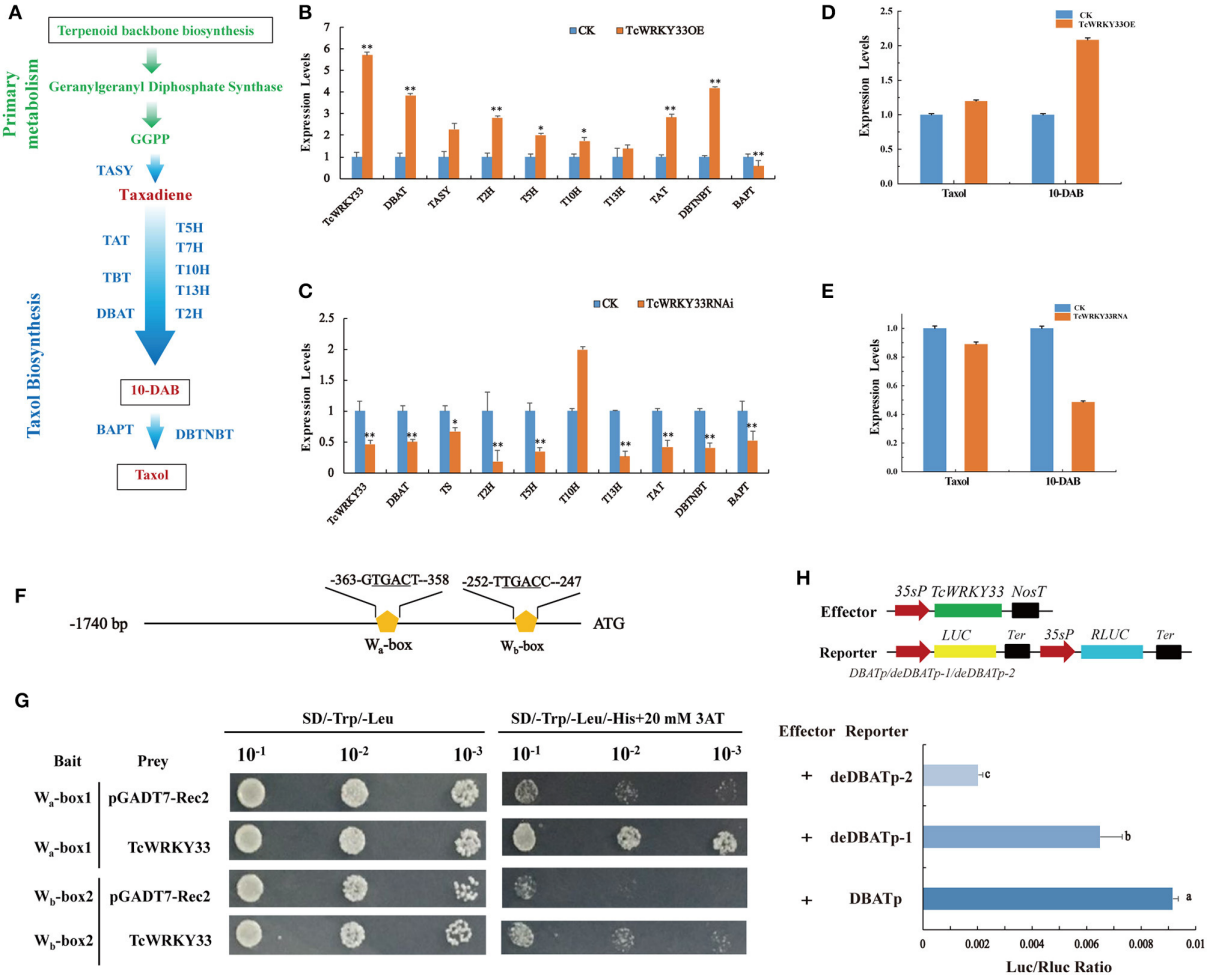
*TcWRKY33* promoted the biosynthesis of taxol and 10-DAB by directly activating *DBAT via* binding with the W-boxes in its promoter. **(A)** A simplified diagram of taxol biosynthetic pathway. *TASY*, taxadiene synthase; *T5H*, taxadiene 5-alpha hydroxylase; *T7H*, 7-alpha-hydroxylase gene; *T10H*, 5-alpha-taxadienol-10-beta-hydroxylase; T13H, 13-alpha-hydroxylase gene; T2H, 2-alpha-hydroxylase gene; TAT, taxadienol acetyltransferase; TBT, taxane 2-alpha-*O*-benzoyltransferase; *DBAT*, 10-deacetylbaccatin III-10-*O*-acetyltransferase; BAPT, phenylpropanoyltransferase; DBTNBT, 3′-*N*-debenzoyltaxol *N*-benzoyltransferase. **(B,C)** The expression levels of taxol biosynthesis genes were quantified in *TcWRKY33* overexpressed and RNA interference leaves by using qRT-PCR. Actin was used as the reference gene, each experiment was conducted three repeats, and totally three biological repeats were used, and the statistics method of *p*-value was Student's *t*-test. Asterisks indicate the significance, *0.01 < *p* < 0.05, and **p < 0.01. **(D,E)** The content of taxol and 10-DAB was quantified in *TcWRKY33* overexpressed and RNA interference transient leaves by HPLC. The content of taxol in the CK group was as a reference. **(F)** Schematic diagram of *DBAT* promoter. The two orange pentagons indicated two W-boxes. **(G)** Yeast one hybrid of *TcWRKY33* and W-boxes in yeast. Related bait vector with each W-box was co-transformed with vacant pGADT7-Rec2 into Y187 as the control. **(H)** Schematic diagrams of the effector vector and reporter vectors. *TcWRKY33* expression plasmid was under the control of the CaMV 35S promoter. Effector and reporter vectors were co-transformed to tobacco leaves, and expression of LUC/Rluc was quantified. Different letters indicate the significant difference (*p* > 0.05) among treatments based on the Duncan's multiple range test of SPSS Statistics 21, SPSS Inc., Chicago, IL, USA.

The biosynthesis of taxol is a complex process and it involves many enzymes (Figure 3A). To identify the function of *TcWRKY33* in regulating the expression of taxol biosynthesis genes, we transiently overexpressed *TcWRKY33* in *Taxus* leaves. Expression levels of taxol biosynthesis genes were examined by using qRT-PCR. The expression of *TcWRKY33* in transformed leaves was 5.72 times greater than that in the control (Figure 3B). Transcriptional levels of *DBAT, TASY*, 2*-alpha-hydroxylase* (*T2H*), *T5H, T10H, TAT*, and *DBTNBT* were 3.85-, 2.27-, 2.81-, 2.01-, 1.74-, 2.84-, and 4.19-fold higher in leaves overexpressing *TcWRKY33* than in controls (Figure 3B). In contrast, the *BAPT* expression was 0.57 times that of controls (Figure 3B).

We also interfered with *TcWRKY33* expression in *Taxus* fresh leaves using transient RNAi technology. Expression levels of *TcWRKY33* in RNAi leaves were 0.47 than that in the control (Figure 3C). Expression levels of taxol biosynthesis genes were measured by using qRT-PCR. Transcriptional levels of most of the taxol biosynthesis genes, including *DBAT, TASY, T2H, T5H, 13-alpha-hydroxylase* (*T13H*), *TAT*, and *DBTNBT*, were 0.50, 0.67, 0.19, 0.35, 0.37, 0.42, and 0.41 times those in the control; however, *T10H* displayed expression levels 2.00-fold those of the control (Figure 3C). These results confirmed that *TcWRKY33* significantly upregulated the expression of taxol biosynthesis genes, especially the key biosynthesis genes *DBAT* and *TASY*. We selected these two genes for further study to explore the regulation mechanisms of *TcWRKY33* on the taxol biosynthesis genes.

### *TcWRKY33* Stimulates the Accumulation of Taxol and 10-DAB

To further determine the effect of *TcWRKY33* on taxol biosynthesis, we measured the content of taxol and 10-DAB (a taxol precursor and product of *DBAT*) using HPLC in *T. chinensis* leaves transiently overexpressing *TcWRKY33* or with *TcWRKY33* expression knocked down through RNA interference. Taxol accumulated to levels in *TcWRKY33*-overexpression leaves of up to 1.20 times higher than that in controls (Figure 3D). Furthermore, 10-DAB levels were up to 2.09 times higher than that in controls (Figure 3D). In *TcWRKY33*-RNA interference leaves, the content of taxol was only 0.89 than that in controls (Figure 3E). The accumulation of 10-DAB was also lower than that in controls by 0.49 (Figure 3E). These results indicate that the *TcWRKY33* transcription factor effectively improved the biosynthesis of taxol. Moreover, *TcWRKY33* seemed to be closely associated with the content of 10-DAB, which is a precursor of taxol produced by *DBAT*.

### *TcWRKY33* Directly Activates *DBAT* by Binding to W-boxes in Its Promoter

Li et al. (2013) discovered two W-boxes (W_a_-box at −363 bp and W_b_-box at −252 bp) within −368 bp of the *DBAT* promoter; these W-boxes are the functional sites of TcWRKY1, a group IIa WRKY factor (Li et al., 2013; Zhang et al., 2018a). *DBAT*, commonly considered one of the key enzymes in taxol biosynthesis, was significantly upregulated by *TcWRKY33*. Thus, *DBAT* appears to be a direct target of *TcWRKY33*. We analyzed the promoter of the *DBAT* gene for potential *cis*-elements regulated by *TcWRKY33* (Figure 3F). Four tandem repeats of the two W-boxes (6 bp) were separately ligated into pHIS2.1 and respectively co-transformed with a *TcWRKY33*-AD fusion vector into yeast Y187 cells. Screening on SD/-Trp/-Leu/-His+20 mM 3-AT medium plates revealed that *TcWRKY33* could bind to the two fragments (Figure 3G).

We further cloned the full-length 383 bp *DBAT* promotor and two progressive deletions removing the W_a_-box and W_b_-box into luciferase reporter vectors and, respectively, co-transformed these with a *TcWRKY33* effector vector into *N. benthamiana* leaves (Figure 3H). LUC/Rluc values were decreased when W-boxes were deleted from the promotors. These results suggest that *TcWRKY33* activates the expression of downstream genes by binding to the two W-boxes in the promoter of *DBAT* (Figure 3H). The LUC/Rluc ratio decreased more when both W-boxes were deleted than when only the W_a_-box was deleted, indicating that the W_b_-box might be the most important binding site for *TcWRKY33* with the best activation activity (Figure 3H).

### *TcWRKY33* Activates the Activator of *TASY* by Binding to W-boxes in the *TcERF15* Promoter

**Figure 4:**
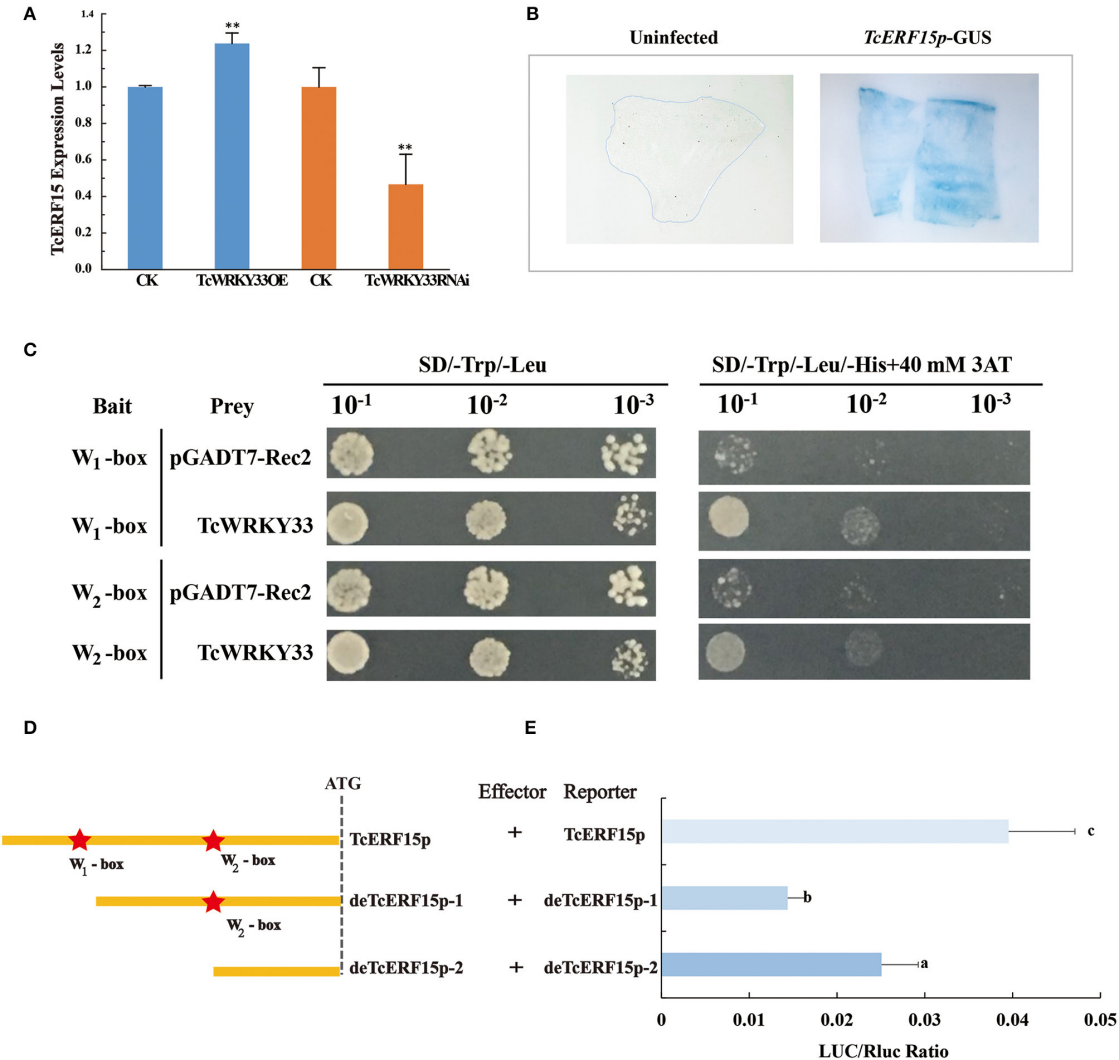
*TcWRKY33* upregulated *TASY* by binding with the promoter of *TcERF15*. **(A)** The expression levels of *TcERF15* were quantified in *TcWRKY33* overexpressed and silenced leaves by using qRT-PCR. Asterisks indicate the significance, **p < 0.01. **(B)** GUS expression in transformed onion epidermis cells after 48 h. The untransformed onion epidermis cells as the negative control, the transformed onion epidermis cells with pBI121-pro_TcERF15_::GUS was dyed blue by X-gluc. **(C)** Yeast one-hybrid analysis of interactions between *TcWRKY33* and W-box. Yeast strains were serially diluted and 2.5 ml of each dilution was plated on synthetic dropout media without Leu and Trp (SD-L-W) plate or synthetic dropout media without Leu, Trp, and His (SD-L-W-H) plus 40 mM of 3-aminotriazole (3AT). **(D)** Schematic diagram of *TcERF15* promoter. *TcERF15* promoter contained two W-boxes that were signed by pentacles. This was the sketch map that the construction of full-length *TcERF15* promotor and two progressive deletions removing the W_1_-box and W_2_-box. **(E)** Effector and reporter vectors were co-transformed, and the expression of LUC/Rluc was detected. Different letters indicate the significant difference (*p* > 0.05) among treatments based on the Duncan's multiple range test of SPSS Statistics 21.

The expression of *TASY*, encoding one of the most important enzymes involved in taxol biosynthesis, was also activated by the *TcWRKY33* transcription factor. However, this gene does not appear to possess a W-box in its promoter. This suggested the presence of an intermediate regulatory factor between *TcWRKY33* and *TASY*. A search of reported regulators identified *TcERF15*, a positive regulator of the *TASY* gene that contains two W-boxes in its promoter (Zhang et al., 2015). After the induction of SA, the expression of *TcERF15* upregulated by 2.66 times at 3 h (Figure 1B), indicating that TcERF15 responded positively to SA signals. When we transiently overexpressed *TcWRKY33* in *T. chinensis* leaves, *TcERF15* expression was slightly but significantly higher by 1.24 times compared with that in controls (Figure 4A). However, *TcERF15* expression was on average 0.47 times that of controls in leaves transiently expressing a *TcWRKY33* RNA interference construct, indicating that *TcERF15* is probably involved in the regulation of *TcWRKY33* (Figure 4A).

We obtained a 715 bp 5′-flanking sequence of *TcERF15*. GUS staining indicated that this sequence could promote the expression of reporter genes and was thus a promoter fragment (Figure 4B). Online tools identified two W-boxes in the *TcERF15* promoter: 5′-TTGACT-3′ at −700 bp (named W_1_-box) and 5′-TTGACG-3′ at −452 bp (named W_2_-box). We quadrupled each 6-bp fragment from the *TcERF15* promoter and mutant fragments and ligated these separately into pHIS2.1; these constructs were, respectively, co-transformed with a *TcWRKY33*-AD fusion vector to detect interaction in a yeast one-hybrid system. Positive clones containing the *TcWRKY33*-AD fusion vector and W-box bait vectors grew on Trp-, Leu-, and His-deficient media with 40 mM 3-AT, whereas control clones did not. This indicated that *TcWRKY33* interacts with W-boxes in the promoter of the *TcERF15* gene (Figure 4C).

The yeast one-hybrid experiment revealed that the two W-boxes of the *TcERF15* promoter were direct targets of *TcWRKY33 in vitro*. For further clarification, we transiently co-transformed full-length of *TcERF15* promoter reporter vectors and two progressive deletion vectors, respectively, along with *TcWRKY33* into *N. benthamiana* leaves (Figure 4D). LUC/Rluc expression was lower when the W_1_-box was deleted compared with expression from the full-length *TcERF15* promoter, indicating that *TcWRKY33* can activate the expression of reporter genes by binding to the W_1_-box. However, when both W-boxes were deleted, the LUC/Rluc ratio was higher than when only the W_1_-box was missing. This suggested that *TcWRKY33* plays a negative regulatory role when interacting with the W_2_-box. The LUC/Rluc expression level was lower than that with the full-length promoter sequence, thus indicating that the activating function of the W_1_-box on *TcWRKY33* is stronger than the negative regulatory role of the W_2_-box (Figure 4E).

## Discussion

Taxol is a precious secondary metabolite that was initially isolated from *Taxus* spp. and is widely used as an anticancer drug (Rowinsky et al., 1990). However, it is found in extremely low levels in *Taxus* trees, making it too expensive to obtain in this manner (Walker and Croteau, 2000). A previous study using TcWRKY overexpression cell lines showed that these transcription factors may have significant effects on the regulation of taxol biosynthesis (Zhang et al., 2018a). SA also improves the accumulation of taxol (Miao et al., 2000). In this study, we isolated an SA-induced WRKY transcription factor, *TcWRKY33*, to explore its function in the biosynthesis of taxol.

Two W-boxes (W_a_-box and W_b_-box) in the promoter of *DBAT* are the main SA-responsive elements (Li et al., 2013). Thus, WRKY transcription factors must be key for transducing SA signals to the *DBAT* gene and possibly the whole taxol biosynthesis pathway. Despite interacting with the two W-boxes, however, TcWRKY1 only responds to MeJA signals (Li et al., 2013). We showed that *TcWRKY33* responds strongly to SA, providing the missing SA-responsive WRKY regulator in taxol biosynthesis. Of seven TcWRKY transcription factors selected from transcriptome data to confirm their function in relation to SA and MeJA signals (Zhang et al., 2018a), only two were upregulated after SA treatment. They were also activated by MJ, which is considered to inhibit the SA response. *TcWRKY33*, therefore, presents a significant advantage as it is only induced by SA signals.

*TcWRKY33* strongly prompted the accumulation of taxol and 10-DAB. Moreover, the expression levels of most taxol biosynthesis genes were increased when *TcWRKY33* was overexpressed. This was particularly true for *DBAT* and *TASY*, which are recognized as the most important enzymes in taxol biosynthesis. The expression levels of these taxol biosynthesis genes were greatly decreased when *TcWRKY33* was silenced. This revealed that *TcWRKY33* functions as an active regulator of taxol biosynthesis by upregulating the expression of most taxol biosynthesis genes, including *DBAT* and *TASY*; this is consistent with other group I WRKY transcription factors functioning as activators (Zheng et al., 2006; Li et al., 2015).

Our yeast one-hybrid and dual-luciferase reporter experiments verified that *TcWRKY33* activates the expression of *DBAT* by interacting with the two W-boxes in its promoter. The W_b_-box showed greater activation activity than the W_a_-box. Furthermore, the levels of the taxol precursor 10-DAB, a product of *DBAT*, also increased by up to 2.09 times after *DBAT* was activated by *TcWRKY33*. This confirms that *TcWRKY33* increases the content of 10-DAB and taxol by directly activating *DBAT* expression. Similar results have been reported in other plants. For example, in *Hylocereus monacanthus*, HmoWRKY40 transcriptionally activates *HmoCYP76AD* by binding to its promoter and is involved in the regulation of pitaya betalain biosynthesis (Zhang et al., 2021). In *Ophiorrhiza pumila*, OpWRKY2 positively regulates the biosynthesis of the anticancer drug camptothecin by binding and activating the central camptothecin pathway gene *OpTDC* (Hao et al., 2021). WRKY transcription factors therefore influence the biosynthesis of secondary metabolites by regulating the expression of key enzyme genes in their biosynthesis pathways. However, *TASY*, one of the most important enzymes in the biosynthesis of taxol, which responds strongly to SA, appears to have no W-box in its promoter. Thus, *TcWRKY33* upregulates its expression levels in other ways.

*TcERF15*, an activator of *TASY*, was activated in leaves transiently overexpressing *TcWRKY33*. Promoter analysis identified two W-boxes in the promoter of *TcERF15*. Yeast one-hybrid and dual-luciferase reporter assays further confirmed that *TcWRKY33* activates *TcERF15* by binding to the two W-boxes. Therefore, *TcWRKY33* directly interacts with the W-boxes in the *DBAT* promoter to activate the expression of *DBAT*. It also increases the expression of *TcERF15* slightly to upregulate *TASY*. Similar results have been observed in *Catharanthus roseus*. For example, CrWRKY1 plays a key role in determining the root-specific accumulation of serpentine by upregulating several key TIA pathway genes, especially *tryptophan decarboxylase*, as well as the transcriptional repressors *ZCT1* (zinc-finger *C. roseus* transcription factor 1), *ZCT2*, and *ZCT3* (Suttipanta et al., 2011). In red-fleshed apples, MdWRKY11 binds to W-boxes in the promoters of *MdHY5, MdMYB10*, and *MdMYB11*, regulating their activity. It also binds to the promoter of *UFGT*, encoding a key structural gene in the anthocyanin biosynthesis pathway, to upregulate the expression of *UFGT*. Hence, MdWRKY11 is involved in *MdHY5*-mediated anthocyanin biosynthesis and regulates the expression of *MdMYB10, MdMYB11* transcription factors, and *UFGT* in apple fruit (Liu et al., 2019). These WRKY transcription factors obviously regulate the biosynthesis of secondary metabolites efficiently and extensively through various regulation patterns. Furthermore, our results show the superiority of *TcWRKY33* in taxol biosynthesis.

**Figure 5:**
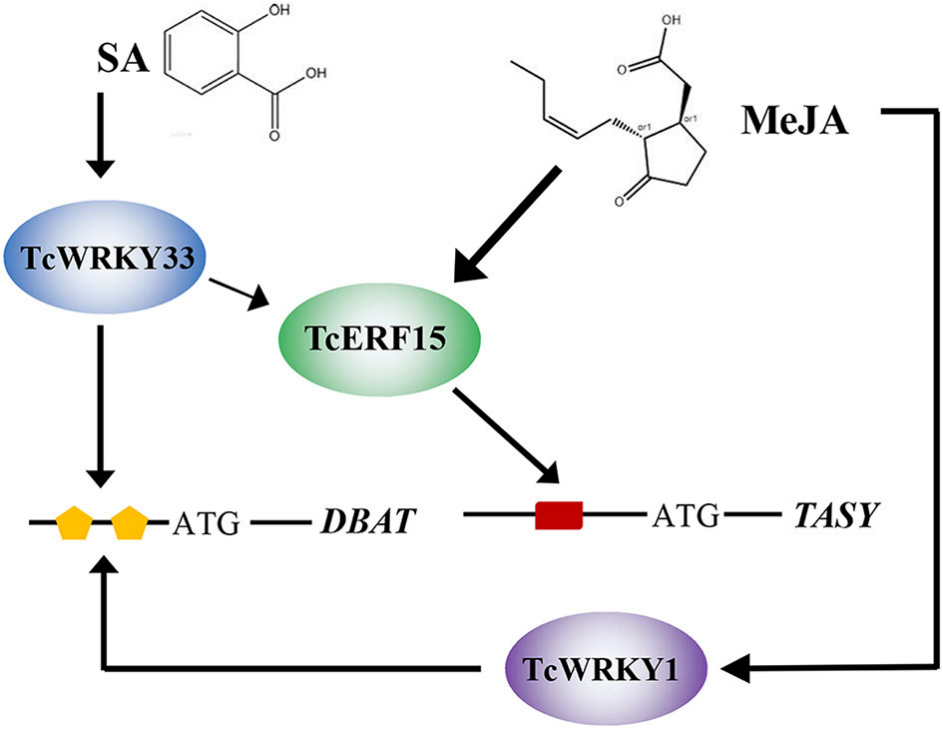
Model summarizing the *TcWRKY33*-mediated regulation of taxol biosynthesis in *T. chinensis*. The lines indicated the regulation. SA and MeJA, plant endogenous hormones; *TcWRKY33* and TcWRKY1, *T. chinensis* WRKY transcription factors; TcERF15, *T. chinensis* ethylene responsive factor; orange pentagon and red rectangle represent W-boxes and GCC-box, *cis*-element in the gene promoter region, respectively; *DBAT* and *TASY*, taxadiene synthases.

*TcWRKY33* clearly responds to SA signals and transmits these SA signals to downstream genes. In addition, *TcWRKY33* appears to regulate downstream genes, such as *DBAT* and *TASY*, in two ways. *DBAT* responds to SA signals, which are transmitted directly to *DBAT via TcWRKY33* interacting with W-boxes. The *TASY* gene is more sensitive to MeJA stimuli, and this mechanism has been clarified extensively (Lin et al., 1996). Additionally, *TASY* expression can be induced by SA, although the related mechanism remains unclear. In this study, we showed that *TcWRKY33* exerts considerable positive effects on *TASY* expression, suggesting that *TcWRKY33* may play important roles in the response of *TASY* to SA. As an activator of *TASY, TcERF15* is a direct target of *TcWRKY33*. *TcWRKY33* activates *TASY* by first regulating *TcERF15* after the induction of SA. Thus, *TcWRKY33* transmits SA signals to *DBAT* and *TASY* to regulate taxol biosynthesis (Figure 5).

## Conclusion

We identified an SA-induced activator in the biosynthesis of taxol and provide references for the role of WRKY transcription factors in the regulation of taxol biosynthesis.

## Data Availability Statement

The original contributions presented in the study are publicly available. This data can be found at: NCBI repository, accession number: MW928850.

## Author Contributions

CF, CZ, and LY conceived the research. YC and CZ designed the study. YC, HZ, MZ, WZ, ZO, and ZP conducted the experiments, including overexpression and RNA interference of *TcWRKY33*, taxanes content detection, subcellular localization, yeast one-hybrid, dual-luciferase reporter experiment, and so on. YC and HZ analyzed the data and wrote the manuscript. All authors read and approved the manuscript.

## Conflict of Interest

The authors declare that the research was conducted in the absence of any commercial or financial relationships that could be construed as a potential conflict of interest.

## Publisher's Note

All claims expressed in this article are solely those of the authors and do not necessarily represent those of their affiliated organizations, or those of the publisher, the editors and the reviewers. Any product that may be evaluated in this article, or claim that may be made by its manufacturer, is not guaranteed or endorsed by the publisher.

## Supplementary Material

Supplementary Table 1 Primers for amplifying genes, constructing vectors, and quantitative real-time PCR.
